# Invisible Ties and Essential Care: Chosen Family Caregivers in the HIV Landscape

**DOI:** 10.3390/geriatrics11040080

**Published:** 2026-07-06

**Authors:** Kristina M. Kokorelias, Luxey Sirisegaram, Dean Valentine, Stephanie Hatzifilalithis

**Affiliations:** 1 Department of Medicine, Healthy Ageing and Geriatrics Program, Sinai Health and University Health Network, Toronto, ON M5G 1X5, Canada; 2 Department of Occupational Science and Occupational Therapy, Temerty Faculty of Medicine, University of Toronto, Toronto, ON M5G 1V7, Canada; 3 Rehabilitation Sciences Institute, Temerty Faculty of Medicine, University of Toronto, Toronto, ON M5G 1V7, Canada; 4 KITE, Toronto Rehabilitation Sciences Institute, 550 University Avenue, Toronto, ON M5G 2A2, Canada; 5 Women’s Age Lab, Women’s College Hospital, Toronto, ON M5S 1B2, Canada; stephanie.hatzifilalithis@wchospital.ca; 6 Dalla Lana School of Public Health, University of Toronto, Toronto, ON M5T 3M7, Canada

**Keywords:** chosen family, HIV, older adults, caregiving, LGBTQ, gender, health equity, social support, chronic illness, aging

## Abstract

Chosen family caregivers assume caregiving roles by choice rather than by blood or legal relation. Chosen family caregivers play a critical yet often invisible role in supporting older adults living with HIV. Unlike traditional family caregivers, many chosen family members are themselves living with HIV or other chronic health conditions, creating unique dynamics of mutual support, resilience, and vulnerability. Older LGBTQ adults are disproportionately reliant on chosen family, often due to stigma, estrangement from biological family, or social marginalization, highlighting distinct challenges in caregiving relationships. Women in chosen family roles frequently experience compounded burdens, balancing emotional, physical, and logistical care responsibilities alongside broader social and structural pressures. Despite their essential contributions, chosen family caregivers remain largely unrecognized within healthcare systems, limiting their access to formal support, respite, and decision-making authority. This commentary synthesizes the existing literature and conceptual perspectives to examine the social, gendered, and health-related dimensions that distinguish chosen family caregiving. It highlights key gaps in recognition and support and outlines implications for policy, research, and practice aimed at strengthening care for older adults living with HIV.

## 1. Introduction

As populations age and antiretroviral therapies extend life expectancy, an increasing number of older adults are living with HIV [1,2,3]. This trend underscores the growing importance of understanding how to support health, well-being, and daily functioning among older adults living with HIV. Within this context, the vital role of caregivers becomes increasingly salient [4]. While much of the caregiving literature focuses on family members defined by biological or legal ties, older adults with HIV frequently rely on chosen family. Chosen caregivers are individuals who are identified by older adults as part of their meaningful support network and who assume caregiving roles by choice rather than by biological, legal, or marital relationship [5]. Chosen family may include friends, partners, neighbours, or community members and extends the traditional concept of family to encompass blended family structures, step-relationships, and other socially or emotionally significant relationships that contribute to care, support, and companionship [5]. These caregivers often navigate complex relational and health dynamics, as many are themselves living with HIV or other chronic conditions [5,6,7].

Chosen family caregiving is particularly salient within LGBTQ communities, where historical stigma, discrimination, and estrangement from biological families make informal, relational networks essential for care [5,6,8]. Women in these roles often bear disproportionate emotional and logistical responsibilities, reflecting broader gendered patterns in caregiving while simultaneously facing unique challenges associated with HIV and aging [9]. Despite their critical contributions, chosen family caregivers remain largely invisible in health systems, with limited access to recognition, formal support, or inclusion in medical decision-making [7].

This paper is a conceptual commentary that draws on existing studies in the peer-reviewed literature, policy discussions, and theoretical perspectives on caregiving, aging, HIV, and LGBTQ health. Consistent with the aims of a conceptual commentary, the literature search was achieved through targeted keyword searches of PubMed/MEDLINE, CINAHL, Scopus, and Google Scholar, with emphasis on English-language literature addressing HIV and aging, LGBTQ/chosen family caregiving, caregiver burden, and geriatric complexity. No formal date restriction was applied, although recent literature was prioritized where available. This paper does not report on primary empirical findings. Instead, it synthesizes and critically examines current knowledge to highlight the distinctive features of chosen family caregiving, identify gaps in recognition and support, and outline key considerations for future research, policy development, and clinical practice. From this body of work, we interpret how chosen family caregiving is constituted through overlapping social, structural, and health-related dynamics, including stigma, gendered expectations, and mutual care within networks of chronic illnesses. This commentary also examines caregiver preparedness and skill-building, support needs in community and social care, caregiver safety and quality of care, collaboration with home healthcare staff, the role of technology in home care, and the positive aspects of caregiving. Building on these insights, we propose that greater recognition of chosen family caregivers must be reflected in practice and policy through inclusive definitions of family, improved access to caregiver support, and formal integration of chosen caregivers into healthcare decision-making processes. The arguments presented are informed by interdisciplinary scholarship and are intended to advance discussion and guide more inclusive approaches to caregiving for older adults living with HIV.

## 2. Recognizing the Invisible Caregivers of Older Adults Living with HIV

### 2.1. Aging with HIV and Geriatric Complexity

HIV carries a legacy of persistent stigma, historical trauma, and social marginalization that profoundly shapes aging and caregiving relationships [10]. The literature suggests that older adults who survived the early HIV epidemic often endured social rejection, medical neglect, and life-threatening illness at a time of intense public fear and discrimination [11]. These experiences continue to influence their social networks, health behaviours, and willingness to engage with healthcare systems. For many, this history makes disclosure of health status, and of non-traditional caregiving arrangements, fraught, particularly when their chosen family caregivers are friends, partners, or community members who are not legally recognized. As individuals age with HIV, these histories intersect with geriatric complexity, including multimorbidity, polypharmacy, functional decline, cognitive change, and frailty, shaping both the intensity and the relational dynamics of caregiving over time [12,13]. At the same time, the practical work of caregiving often expands from HIV-related support to ongoing care coordination: managing changing medication regimens and potential drug–drug interactions, monitoring for functional or cognitive shifts, supporting adherence and safety, and accompanying care recipients through transitions across settings (e.g., hospital-to-home, rehabilitation, home care, or long-term care), where continuity depends on timely communication and advocacy [14,15,16]. As a result, caregiving relationships may operate largely in private, invisible, or improvised ways, with caregivers managing medical and emotional responsibilities while navigating the constraints of systemic invisibility and limited formal authority during clinical encounters and discharge planning.

### 2.2. Chosen Family Caregiving in HIV Care

Unlike traditional family caregivers, chosen family caregivers enter caregiving relationships by negotiation and mutual commitment rather than obligation [5]. These relationships are not simply defined by shared history but by actively forged solidarity, where individuals come to care for one another through experiences of social marginalization, shared struggle, or reciprocal trauma [5]. Within many communities, particularly among populations historically affected by stigma and health inequities, chosen caregiving networks function as spaces of resilience, safety, and affirmation [17]. The available literature supports that the caregiving bond is often maintained through ongoing negotiation of roles, expectations, and boundaries, reflecting a relational ethic grounded in trust, respect, and mutual recognition of each other’s needs, values, and autonomy [17,18]. Many chosen family caregivers are themselves managing chronic health conditions, including HIV, which creates a dynamic of mutual support but also amplifies vulnerability [18]. Caregiving in these contexts is often fluid, with responsibilities shifting over time based on health status, availability, and relational dynamics [18]. This flexibility, while adaptive, also presents challenges. Health systems are typically designed to recognize formal, legally defined caregivers, limiting the ability of chosen family members to access support such as respite care, caregiver allowances, or involvement in clinical decision-making [7]. Consequently, many chosen family caregivers operate in invisibility, providing essential care without recognition, guidance, or protection [6,7].

Older adults living with HIV frequently face layered marginalization, ageism, HIV-related stigma, and, for many, homophobia or transphobia, which can lead to estrangement from biological families or limited social support [17,19]. For many long-term survivors, contemporary aging and caregiving cannot be understood apart from the early HIV epidemic, when diagnosis was widely experienced as a near-certain death sentence and care unfolded amid profound stigma, institutional neglect, and repeated bereavement within social networks. Qualitative work with long-term survivors describes enduring psychological and social sequelae of that period, including survivor guilt, anxiety, depression, and a persistent sense of uncertainty that can reverberate into later life and shape how support is sought, accepted, or refused [20]. These historical conditions have lasting implications for healthcare engagement: experiences of discrimination and dismissiveness in earlier decades can contribute to heightened vigilance about disclosure, reluctance to involve non-legal care partners in clinical settings, and mistrust of institutions, especially when older adults anticipate judgment related to HIV, sexuality, gender identity, substance use, or poverty [3]. In this context, chosen family becomes not only the primary source of emotional care but also an essential buffer against social isolation and exclusion [17]. Many older adults with HIV also contend with a lifetime of structural inequities, including housing instability, poverty, and barriers to consistent healthcare access that can be compounded by comorbidities and age-related functional decline [21]. Chosen family caregivers often serve as advocates, care coordinators, and system navigators, managing medical appointments, medication adherence, and interactions with healthcare providers. Their role is uniquely shaped by the ongoing social and medical stigma of HIV, making their support indispensable for both the health and dignity of older adults living with HIV.

Positive aspects of chosen family caregiving should also be recognized [22]. Many caregivers describe meaning, reciprocity, closeness, identity affirmation, and a sense of purpose through caregiving relationships [22]. In chosen family networks, care can strengthen trust, deepen belonging, and sustain communities that have historically been excluded from formal systems [23]. Recognizing these strengths is important because it avoids framing caregiving only as a burden and instead reflects the full range of caregiver experience.

### 2.3. Gendered Caregiving Burden

Gender plays a critical role in shaping caregiving experiences. Women in chosen family caregiving roles often carry the brunt of emotional, practical, and logistical responsibilities, reflecting entrenched societal expectations of women as caregivers [9,24]. For older adults living with HIV, this burden may be compounded by caregiving for partners, friends, or community members who are also managing chronic illness, navigating stigma, or coping with social isolation [25,26]. These gendered expectations intersect with broader social and structural inequities as well as the clinical realities of aging with HIV, where women caregivers living with HIV may simultaneously manage their own health needs while providing care to older adults living with HIV, often navigating multiple, overlapping medical and psychosocial challenges. They routinely coordinate medical appointments, troubleshoot antiretroviral and other medication regimens for themselves and their care recipients, and respond to geriatric syndromes that become more common with age [26]. Women also frequently balance multiple roles, including employment, parenting, and their own health management, which can increase stress and risk for burnout [27]. Within LGBTQ+ networks, women often experience compounded caregiving burden at the intersection of multiple systems of inequality, where historical marginalization of sexual and gender minorities intersects with HIV stigma, ageism, and broader structures of social disadvantage [3,7,9,21]. These overlapping forms of structural inequity can create a “matrix of dominance” that shapes how caregivers access, navigate, and experience the health system, often making engagement in care more complex, less affirming, and more emotionally and administratively demanding. Recognizing these layered, clinically grounded challenges is essential for designing targeted interventions, such as geriatric-informed HIV care, integrated medication management, and caregiver-inclusive care planning that support both caregivers and care recipients.

### 2.4. Caregiver Preparedness, Education, and Safety

Chosen family caregivers often take on complex care tasks without formal preparation. While this is not unique to other family caregivers, their role may not be automatically acknowledged by health systems. As a result, they may be excluded from discharge teaching, care conferences, and written instructions, even when they are the person most involved in day-to-day care [7,28]. In this context, education is not only about skill building but also about helping chosen caregivers gain the legitimacy and access needed to provide safe and consistent care.

### 2.5. System Redesign and Call to Action for Healthcare Policy and Practice

The literature is consistent in showing that caregiving for older adults living with HIV is often embedded in chosen family networks forged through histories of stigma, loss, and social exclusion, and sustained through negotiated reciprocity and ongoing flexibility of roles [5,17,18]. It also highlights that many chosen caregivers share similar vulnerabilities such as living with HIV or other chronic conditions and that women disproportionately shoulder the emotional and logistical labour that keeps care functioning [25,26]. Yet what remains under-recognized in routine practice is the structural mismatch between how care is actually delivered and how caregiving is formally recognized: healthcare systems frequently treat chosen caregivers as peripheral or unofficial, even when they serve as care coordinators, advocates, medication supports, and system navigators [6,7]. This matters for care delivery because exclusion disrupts information flow, weakens continuity and adherence support, impedes shared decision-making, and shifts the burden onto caregivers without protection, training, or respite, thereby increasing risk of burnout and avoidable care failures. Community support may include respite services, peer support groups, caregiver education sessions, navigation help, psychosocial counseling, practical home support, and assistance connecting to housing, income, and transportation resources. On this basis, the need for system redesign logically follows from the evidence because chosen caregivers already underpin day-to-day clinical management and psychosocial stability for many older adults living with HIV; policy and practice must align with reality by formally recognizing chosen family, integrating them into care planning processes, and addressing gendered burden through equitable support.

Supporting chosen family caregivers requires intentional policy and practice shifts. Healthcare systems must broaden definitions of family and caregivers to include relationally defined, non-legal caregivers. Chosen family caregiving should be understood as part of a shared care network that includes home care nurses, personal support workers, therapists, case managers, and physicians. Collaboration works best when roles are clear, communication is routine, and caregivers are included in care planning with consent [29]. Home care nurses can support caregiver preparedness by reinforcing teaching, checking understanding, identifying safety risks in the home, and helping coordinate changes in care needs [28]. When chosen caregivers are excluded from communication, medication errors and avoidable transition problems may become more likely [30]. Training should include skills-based preparation for caregiving tasks, coordination with home care nurses, appropriate use of digital tools, and guidance on caregiver safety and escalation pathways [31]. Policies should enable chosen family members to access caregiver support, training, and inclusion in clinical decision-making. For example, HIV clinics and affiliated services should implement a standardized “chosen caregiver” designation in intake and follow-up processes (including explicit consent for information sharing and involvement in care planning), so that the person doing day-to-day coordination can reliably participate in visits, transition planning, and post-discharge follow-up. Second, care models should embed routine, brief geriatric screening, covering function, cognition/mentation, mobility/falls, and medication review, as these are common vulnerabilities in older adults with HIV and directly shape the type and intensity of caregiving required [25]. Interventions should also address gendered burdens, offering tailored respite, counseling, and financial support to women who disproportionately shoulder caregiving responsibilities. Research must further explore the experiences, needs, and outcomes of chosen family caregivers, including longitudinal studies that examine the interplay of caregiving, chronic illness, and social marginalization. Capturing these dynamics will provide the evidence base needed to inform policies that recognize and support chosen family caregivers as critical partners in the care of older adults living with HIV.

The literature reviewed here indicates that chosen family members frequently perform this coordination work (e.g., medication support and reconciliation, appointment management across multiple clinicians, monitoring function/mentation, and advocacy during hospital-to-home transitions) yet remain inconsistently visible in documentation and are excluded from routine communication pathways when they lack legal status. On this basis, the policy and practice direction that follows from the evidence is not a generalized call for “culture change” but a targeted set of workflow-level reforms. Similarly, the central message of this commentary is that attention to chosen caregivers cannot remain confined to research discourse alone. The evidence points to a clear need for health systems to move beyond recognition toward meaningful action that supports relationally diverse caregiving networks. Ensuring equitable care for older adults living with HIV requires structural and cultural transformation within healthcare systems so that inclusion of chosen caregivers is not treated as an optional enhancement but as a necessary component of high-quality, person- and family-centered care. Without intentional change, existing models risk reinforcing inequities that continue to exclude or marginalize the very networks that sustain many older adults living with HIV. What is needed now is the translation of knowledge into practice through policy reform, workforce education, and care delivery redesign that normalizes the involvement of chosen caregivers, supports their roles, and acknowledges their contribution to health and well-being across the care continuum.

## 3. Conclusions

Chosen family caregivers represent an essential yet often overlooked component of care for older adults living with HIV. As highlighted in this commentary, these caregiving relationships are shaped by distinct social, gendered, and health-related dynamics, including mutual care arrangements, reliance on informal networks, and the enduring effects of stigma and marginalization. The disproportionate caregiving burden experienced by women within chosen family networks further underscores the importance of adopting an intersectional lens in understanding caregiving experiences.

Although the existing literature provides important insights into these dynamics, gaps remain in how chosen family caregivers are recognized, supported, and integrated into healthcare systems. Greater conceptual and empirical attention is needed to better understand their experiences and to develop inclusive policies and practices. Efforts to formally acknowledge chosen family caregivers, expand access to support services, and ensure their inclusion in care planning and decision-making processes are critical steps toward improving health and well-being outcomes for both caregivers and care recipients.

By synthesizing current knowledge, this commentary underscores the need for more inclusive frameworks that move beyond traditional definitions of family and caregiving. Advancing research, policy, and practice in this area is essential to addressing inequities and strengthening support for older adults living with HIV and those who care for them.

## Data Availability

No new data were created or analyzed in this study. Data sharing is not applicable to this article.

## Abbreviations

The following abbreviations are used in this manuscript:

HIV	Human immunodeficiency virus
LGBTQ	Lesbian, gay, bisexual, transgender, and queer
LGBTQ+	Lesbian, gay, bisexual, transgender, queer, and additional sexual and gender identities
